# Y chromosome functions in mammalian spermatogenesis

**DOI:** 10.7554/eLife.67345

**Published:** 2021-10-04

**Authors:** Jeremie Subrini, James Turner

**Affiliations:** 1 Sex Chromosome Biology Laboratory, The Francis Crick Institute London United Kingdom; Duke University United States; The University of Hong Kong Hong Kong

**Keywords:** Y chromosome, fertility, spermatogenesis

## Abstract

The mammalian Y chromosome is critical for male sex determination and spermatogenesis. However, linking each Y gene to specific aspects of male reproduction has been challenging. As the Y chromosome is notoriously hard to sequence and target, functional studies have mostly relied on transgene-rescue approaches using mouse models with large multi-gene deletions. These experimental limitations have oriented the field toward the search for a minimum set of Y genes necessary for male reproduction. Here, considering Y-chromosome evolutionary history and decades of discoveries, we review the current state of research on its function in spermatogenesis and reassess the view that many Y genes are disposable for male reproduction.

## Introduction

In therian mammals, with some exceptions, whether an embryo will develop into a male or a female is defined by the presence or absence of a Y chromosome (Capel, 2017). Males bear an X and a Y, whereas females carry two X chromosomes. This is the most fundamental genetic difference between the two sexes and has been the subject of numerous studies.

Historically, the Y chromosome’s biological function has been misunderstood. From the 1950s, it was considered to be a genetic wasteland as studies of human pedigrees were only uncovering traits with autosomal or X-linked inheritance (Stern, 1957). In 1959, it was shown that the male-determining gene is Y-linked, but this was considered an exception on an otherwise functionally inert chromosome (Ford et al., 1959; Jacobs and Strong, 1959). When transcription units were first discovered on the Y chromosome (Agulnik et al., 1994; Arnemann et al., 1991; Page et al., 1987; Reijo et al., 1995; Salido et al., 1992; Sinclair et al., 1990), they were thought to be inactive vestiges of their former autosomal ancestors (Marshall Graves, 1995). More recently, the ‘impending demise’ theory hypothesised a continuous loss of Y-protein-coding genes predicting the eventual loss of the Y chromosome (Aitken and Marshall Graves, 2002; Marshall Graves, 2004). We now know that the view of the Y as a disappearing genetic desert is incorrect. Decades of research have proved that besides controlling male gonadal sex determination, the Y chromosome is crucial for initialisation, maintenance, and completion of spermatogenesis.

In this review, we first describe the evolutionary history of the X-Y chromosome pair, then use it as a paradigm with which to understand how the Y chromosome has become functionally specialised across mammals. Focusing on humans and mice, we discuss the early evidence that the Y chromosome is more than just a sex switch, and the subsequent efforts to discover the Y genes involved in spermatogenesis. We then highlight how experimental limitations have impacted progress in the field and propose approaches to enrich our understanding of Y-chromosome function.

### Evolution of the mammalian sex chromosomes

**Figure 1.**
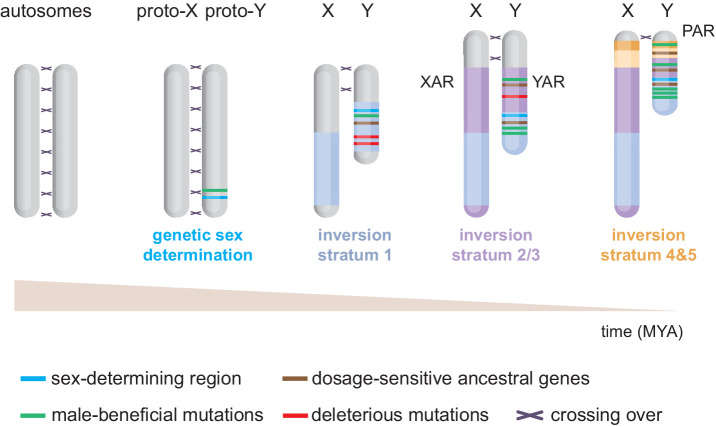
Evolution of the eutherian sex chromosomes. The X and Y chromosomes evolved from a pair of autosomes. First, the testis-determining gene *Sry* evolved on the proto-Y. Then, successive stratification events occurred, wherein X-Y meiotic recombination arrests along specific regions of the Y, likely due to inversions. The first stratum (i.e. discrete non-recombining region) includes Sry and originated in the last common therian ancestor ~166 million years ago (MYA). After the split from marsupials, the X/Y-added regions (XAR/YAR) fused from an autosome to the sex chromosomes in the eutherian ancestor. A second stratification event occurred independently in both marsupial and eutherian ancestors ~ 97–117 MYA. Concomitantly, in eutherians, a third stratum was formed encompassing the YAR. A fourth and fifth stratum evolved in the ancestor of old-world monkeys ~ 25–44 MYA. Recombination now only occurs within the pseudoautosomal region (PAR).

The mammalian heteromorphic X and Y chromosomes have evolved from an ancestral pair of autosomes (Figure 1). This seminal concept, like many principles governing sex chromosome evolution, was hypothesised by Ohno, 1967. Henceforth, breakthroughs in Y-chromosome sequencing and assembly, which were long hindered by the Y’s repeat-rich nature and homology to the X chromosome, have been crucial to understand sex-chromosome evolution (Bellott et al., 2014; Hughes et al., 2012; Hughes et al., 2010; Li et al., 2013; Skaletsky et al., 2003; Soh et al., 2014). We now know that divergence into a ‘proto-Y’ started following mutations of *Sox3*, creating the sex-determining region *Sry* gene (Foster and Graves, 1994; Marshall Graves, 1998). The evolution of *Sry*-specific sex determination is thought to have happened around 160–166 million years ago after the split between therians and monotremes (egg-laying mammals) (Potrzebowski et al., 2008; Veyrunes et al., 2008), and prior to the split between marsupials (metatherians) and eutherians (Luo et al., 2011).

The male-specific inheritance of the proto-Y exposed it to unique evolutionary forces (Larson et al., 2018). Notably, it facilitated selection and accumulation of male-beneficial mutations in the vicinity of *Sry* (Figure 1). The linkage between *Sry* and male-beneficial loci in turn created selective pressure to supress X-Y crossing over. Eventually, multiple discrete inversions on the Y supported and amplified meiotic-recombination arrest in specific regions, or strata (Figure 1; Bellott et al., 2014; Cortez et al., 2014; Lahn and Page, 1999). Stratification expanded the non-recombining male-specific region at the expense of the recombining portion of the chromosome known as the pseudoautosomal region (PAR) (Figure 1). In addition to these strata, (retro)transpositions/translocations from autosomes have occurred more recently in various mammalian lineages (Bellott et al., 2014; Cortez et al., 2014; Hughes et al., 2012).

Importantly, the loss of recombination associated with stratification allowed accumulation of Y-linked loss-of-function mutations, triggering chromosome-wide gene decay and amplification of repetitive DNA (Bachtrog, 2013). As a mechanism to maintain male fitness, large deletions of deleterious and non-functional DNA regions occurred within each non-recombining stratum (Bachtrog, 2013; Hughes and Page, 2015). Although 98% of the ancestral human X-genes survived (Mueller et al., 2013), only 3% persisted on the human Y (Bellott et al., 2014; Skaletsky et al., 2003). However, although the Y experienced rapid decay early in evolution, degeneration then dramatically slowed down, with genes having survived this ‘purge’ showing stability (Bellott et al., 2014; Cortez et al., 2014; Hughes et al., 2012; Hughes et al., 2010; Hughes et al., 2005). For instance, of the 18 ancestral genes present in the ~97 million year old eutherian ancestor (Bellott et al., 2014), humans retain 14, bull 13, chimp 11, and mouse 9. In humans, no Y genes appear to have been lost in the last 44 million years.

In addition, the longevity of certain X-Y gene pairs has been convergent between eutherians and marsupials. Indeed, one stratum independently evolved in both therian lineages (Figure 1). Two genes on this stratum have convergently survived, suggesting that Y gene decay is not stochastic (Bellott et al., 2014). Altogether, it is likely that X-Y gene-pair retention did not occur at random. Generally, it is thought that two evolutionary strategies have protected a specific set of Y genes: maintenance of ancestral X-Y gene pairs which are sensitive to transcriptional dosage; or retention and amplification of testis-biased gene families.

### Evolutionary forces and functional specialisation of the Y chromosome

Whereas X-chromosome content has remained mostly unchanged over evolutionary time, the Y chromosome has experienced extensive genetic decay. As a result, females carry two copies of the numerous X-linked genes, whereas males carry a single copy. This creates an imbalance between X and autosome transcriptional dosage (X:A ratio) in males as well as between sexes (Table 1). This led to the evolution of dosage-compensation mechanisms, as hypothesised by Ohno, 1967. Firstly, twofold X-chromosome upregulation in both males and females re-established X:A equilibrium in males (Table 1; Deng et al., 2014). However, without other mechanisms, the X:A ratio in females would be two. This was prevented by the evolution of X-chromosome inactivation, a process by which one X is transcriptionally silenced in females, bringing the overall X-output level to that of the autosomes (Table 1; Brockdorff et al., 2020; Loda and Heard, 2019; Lyon, 1961).

**Table 1.** Dosage compensation of the sex chromosomes. Without dosage compensation, the transcriptional output of each cell is 0.5 for individual chromosomes, and one for chromosome pairs. X-chromosome upregulation (XCU) doubles each X-chromosome’s output (shown in red). X-chromosome inactivation (XCI) silences output of one X in females (shown in blue).

	No compensation	XCU	XCI
female	AA = 1XX = 1	AA = 1 XX = 2	AA = 1XX = 1
male	AA = 1XY = 0.5	AA = 1XY = 1	AA = 1XY = 1

**Figure 2.**
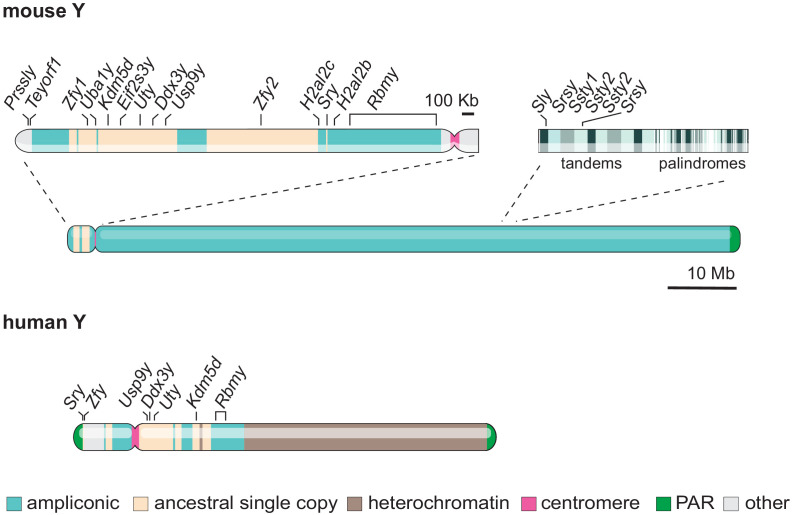
Gene content and structure of the mouse and human Y chromosome. The short arm of the mouse Y chromosome (Yp) has retained ancestral non-ampliconic genes. The mouse Y chromosome long arm (Yq) contains alternating tandem and palindromic repeats of ampliconic core blocks throughout. Ampliconic core blocks encompass rodent-specific gene clusters. The human Y shares seven ancestral protein-coding genes with the murine Y (labelled). Whereas the human Y chromosome is roughly 60 % heterochromatic, the mouse Y chromosome is 99.9% euchromatic (Skaletsky et al., 2003; Soh et al., 2014). The 10 Mb scale bar applies to both mouse and human Y chromosomes.

X-upregulation and X-inactivation can ultimately compensate for genes lost on the Y. However, as an X-Y gene pair loses its Y-copy due to decay, it goes through a transition period during which there will be gene-dosage imbalance. During that intermediate state, the pair has a non-functional Y gene and a non-compensated X gene. For haploinsufficient genes, with function sensitive to their expression dosage, this transition state would be disadvantageous and selected against. As such, dosage-sensitive Y genes have been under strong selective pressure to survive. For instance, studies indicate that Y ancestral genes that share ubiquitous housekeeping regulatory functions with their X homologue, which would be very sensitive to change in expression levels, have preferentially survived on the Y (Bellott et al., 2014; Cortez et al., 2014; Jegalian and Page, 1998; Lahn and Page, 1997). Indeed, the annotations of remaining X-Y pairs link them to the sensitive regulation of histone lysine demethylation, stem-cell self-renewal, splicing, translation initiation and deubiquitylation (Bellott et al., 2014; Lahn and Page, 1997). A key prediction is that if an X-Y pair survives and maintains ancestral gene expression and function, the X copy should escape X-inactivation in females to avoid dosage imbalance. Indeed, the study of allele-specific expression in human, mouse and opossum has revealed that a higher proportion of X-linked genes with a surviving Y homologue escape X-inactivation, compared to genes with no Y homologues or with those showing signs of functional differentiation (Bellott et al., 2014; Cortez et al., 2014). The strict dosage requirement of sex-linked genes can be further inferred from individuals with Turner syndrome (XO), who only have a single full X chromosome (Gravholt et al., 2019). Indeed, human X monosomy is associated with poor embryonic viability and individuals surviving into adulthood tend to be mosaic for at least part of the second sex chromosome. This illustrates the severity of X-X or X-Y dosage imbalance. Interestingly, Turner syndrome is less severe in mice, even in fully XO animals (Burgoyne et al., 1983b; Burgoyne et al., 1983a; Burgoyne and Baker, 1981). This could be in part because mice have fewer surviving Y-linked ancestral genes (9 vs 17 in humans) (Figure 2).

Another strategy thought to have protected Y-genes from regulatory and structural decay is the specialisation towards functions in male fertility. Indeed, ancestral, single-copy genes have sometimes diverged towards male-specific reproductive functions. The expression of some Y genes became almost exclusively limited to the testis, gradually losing expression in other organs to become testis-biased (Jegalian and Page, 1998; Mitchell, 2000). In parallel, the Y chromosome has gained additional testis-biased genes through (retro)transposition and translocation events from autosomes (Burgoyne, 1998). Importantly, some recently acquired testis-biased X/Y gene homologues have been massively amplified, forming tandem and palindromic repeats (Hughes et al., 2020; Rozen et al., 2003; Skaletsky et al., 2003; Soh et al., 2014; Trombetta and Cruciani, 2017; Figure 2). Following amplification, repeats have been maintained and homogenised through intra-chromosomal recombination (Trombetta and Cruciani, 2017). This recombination within the Y can either remove or fix mutations, beneficial or deleterious, and male-advantageous mutations would have then been selected for over time. Overall, amplification seems to have contributed to the longevity of some testis-biased Y genes and it has occurred independently in many lineages. In mice, bull and likely other mammals, amplification of sex chromosomes is thought to have been driven by the presence of antagonistic meiotic drivers (selfish genes) on the X and Y, each competing for transmission to the next generation (Cocquet et al., 2012; Ellis et al., 2011; Hughes et al., 2020; Soh et al., 2014).

Altogether, evolutionary insights have shown that surviving mammalian Y genes tend to fall into two categories: testis-specialised fertility genes or dosage-sensitive ubiquitous housekeepers (Lahn and Page, 1997). This is unlike mammalian autosomes which harbour genes with highly variable biological functions and expression patterns. However, despite the apparent specialisation of the Y chromosome, its exact somatic and fertility functions remain to be fully dissected. Particularly interesting are the functions of the Y chromosome in the context of spermatogenesis, which will be discussed in the rest of this review.

### Mammalian spermatogenesis

**Figure 3.**
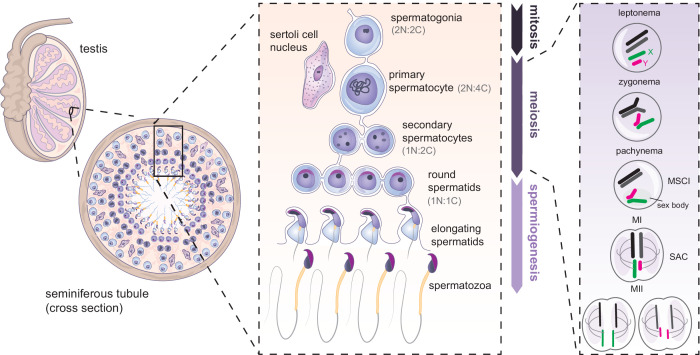
Mammalian spermatogenesis in seminiferous tubules of the testis. Spermatogenesis occurs in three main phases: mitosis, meiosis and spermiogenesis. First, undifferentiated spermatogonial cells undergo multiple mitotic divisions. Subsequently, germ cells commit to meiosis, undergoing meiotic DNA replication (becoming 2 N:4 C) synapsis and recombination between homologues (shown in black and dark gray), and silencing of the X (green) and Y (magenta) chromosomes. Spermatocytes then undergo two rounds of cell division (becoming 1N:1C). The resulting round spermatids later undergo spermiogenesis, further specialising into mature spermatozoa. N = number of chromosomes, C = number of chromatids. SAC: spindle assembly checkpoint.

Spermatogenesis allows continuous formation of gametes from spermatogonial stem cells (Figure 3). It occurs in cycles and can broadly be divided in three phases: mitosis, meiosis and spermiogenesis (Figure 3). All stages happen in the epithelium of the seminiferous tubules of the testis. As cells progress through development, they migrate away from the basal seminiferous membrane towards the lumen (Figure 3). The spermatogenic cycle has been mostly studied in rodents but the mechanisms are believed to be well-conserved across mammals. A major difference seems to be in developmental tempo. In mouse, one cycle can be dissected into 12 stages occurring over 8.6 days, and four cycles (35 days) are required to complete spermatogenesis, from spermatogonial stem cells to mature spermatozoa (de Rooij, 2017; Oakberg, 1956a; Oakberg, 1956b). In humans, one spermatogenic cycle is completed every 16 days, and ~72 days are needed for full differentiation of spermatogonial stem cells into spermatozoa (Muciaccia et al., 2013).

Early in embryogenesis, at embryonic day (E) 6.25 in mouse and E12 in humans, the primordial germ cells are specified and subsequently migrate to the genital ridge (Kurimoto and Saitou, 2019; Sybirna et al., 2019; Tang et al., 2016). The early bipotent gonad is formed at E10.5–11.5 in mouse and week six in humans. *Sry* then triggers a signalling cascade resulting in the supporting cell lineage differentiating into Sertoli cells (Capel, 2017; Kurimoto and Saitou, 2019). Sertoli cells then direct adoption of the testicular state within the gonad. Germ cell proliferation will then occur until developmental arrest at E13.5 in mouse and after week 20 in human (Kurimoto and Saitou, 2019; Tam and Snow, 1981).

After birth, at around postnatal day (P) three in mouse and 2 months in humans, germ cells resume cell division, giving rise to undifferentiated spermatogonial cells that are found at the basal compartment of seminiferous tubules. Amongst this cell population lie spermatogonial stem cells that can both self-renew and multiply through mitotic divisions. The spermatogonial differentiation and proliferation through mitosis marks the starting point of spermatogenesis. It is a specific subset of spermatogonial cells, referred as type-B, that are now ready to undergo meiotic DNA replication and differentiate into pre-leptotene spermatocytes (de Rooij, 2017; Wu et al., 2009).

Male meiosis is initiated in a synchronous manner during puberty, at P10 in mouse and ~13 years in humans. Meiosis occurs through two rounds of cell division. During prophase I, which takes 2 weeks in mouse, diploid primary spermatocytes (2 N:4 C) enter leptonema, zygonema, pachynema, and diplonema successively (Figure 3; Baudat et al., 2013; Oakberg, 1956b). At leptonema, meiotic DNA double-strand breaks are formed. At zygonema, homologous chromosomes synapse and form bivalents (Figure 3). This allows crossing over via reciprocal recombination between homologues. Unlike autosomes, the X and Y are transcriptionally silenced at pachynema through the process of meiotic sex chromosome inactivation (MSCI) (Turner, 2015) and compartmentalised into a specialised chromatin domain called the sex body (Figure 3; McKee and Handel, 1993; Solari, 1974). MSCI failure leads to pachytene arrest and apoptosis. MSCI persists throughout diplonema, when homologous chromosomes start to separate (Burgoyne et al., 2009). The molecular mechanisms and roles of MSCI in fertility have been reviewed previously (Turner, 2015).

After prophase I, metaphase I takes place and chromosome homologues are lined up along the metaphase plate. At this stage, the spindle assembly checkpoint (SAC) ensures proper chromosome alignment and spindle attachment (Figure 3; Lane and Kauppi, 2019). Upon satisfaction of the SAC, homologues finally separate to opposite poles of the meiotic cells and the first division occurs with cytokinesis, giving rise to haploid secondary spermatocytes (1 N:2 C). The second meiotic division separates sister chromatids, leading to the formation of round spermatid cells (1 N:1 C) (Figure 3). These haploid cells then undergo a complex post-meiotic differentiation programme called spermiogenesis (Figure 3).

Spermiogenesis is characterised by the emergence of unique sperm-specific morphological features and by dramatic epigenetic reprogramming (Bao and Bedford, 2016; O’Donnell, 2014). Importantly, DNA of differentiating spermatids undergo increasing compaction in the nucleus (O’Donnell, 2014). This is achieved notably through near genome-wide replacement of histones with protamines. Protamines permit an up to 20-fold increase in chromatin condensation (Balhorn, 2007). Structurally, the acrosome starts to form over the anterior part of the spermatid nucleus and the flagellum, the propeller of the sperm, elongates on the posterior side (Figure 3). Additionally, the cytoplasm is transported along the spermatid tail and finally disposed (O’Donnell, 2014). Interestingly, most X/Y-linked genes remain repressed throughout spermiogenesis (Greaves et al., 2006; Namekawa et al., 2006; Sin et al., 2012; Turner et al., 2006), but some are re-activated (Hendriksen, 1999; Hendriksen et al., 1995; Mueller et al., 2013; Mueller et al., 2008; Wang et al., 2005). By the end of this post-meiotic stage, sperm are freed into the lumen of the tubule.

### The Y chromosome: more than just a sex switch

The first evidence of the Y chromosome having any biological function arose from studies of mice (Welshons and Russell, 1959) and humans with Turner (XO) or Klinefelter (XXY) syndrome (Ford et al., 1959; Jacobs and Strong, 1959). These reports showed that in eutherian mammals, sex was determined by the presence/absence of the Y, independent of the number of X chromosomes. Later research on sex-reversed men; rare individuals which are phenotypically males but have an XX karyotype, narrowed down the search for the male-determining region to the short arm of the Y chromosome. For instance, using DNA hybridisation with several Y-DNA probes, it was showed that these XX males carried translocated DNA from the Y chromosome on one of their X chromosome (Guellaen et al., 1984; Page, 1986; Page et al., 1987; Page et al., 1985). As additional XX males were studied and the number of markers increased, the candidate region was narrowed to 35 kb containing *SRY* (Sinclair et al., 1990). In the span of 2 years, the evidence for *SRY* being the testis-determining factor became undeniable. The mouse orthologue was cloned and found to be expressed in specific somatic cells of the embryonic gonad at the time of testis differentiation (Gubbay et al., 1990; Koopman et al., 1990). It was also shown that both sex-reversed XY mice and women had mutations in *Sry* (Berta et al., 1990; Jäger et al., 1990; Lovell-Badge and Robertson, 1990). Finally, it was shown that, in mouse, *Sry* alone is sufficient to induce testicular development (Koopman et al., 1991), as XX mice transgenic for *Sry* developed as males.

*Sry* is a transcription factor of the SOX family that encodes a protein with an high mobility group (HMG)-box DNA binding domain (Table 2; Kashimada and Koopman, 2010; Figure 2). Much of what is known about the function of *Sry* in testis determination result from mouse studies. During murine embryogenesis, *Sry* upregulates *Sox9* in Sertoli cell precursors between E11.5 and E12.5 (Kashimada and Koopman, 2010). SOX9 then activates fibroblast growth factor 9 (*Fgf9*) in a feedforward loop, which represses *Wnt4* and the female pathway that would otherwise trigger ovary development (Capel, 2017; Kashimada and Koopman, 2010). Importantly, it has been shown that precise regulation of levels and onset of *Sry* expression is key for Sertoli cell differentiation. Indeed, minor alteration in amount and timing of *Sry* expression leads to sex-reversal phenotypes (Kashimada and Koopman, 2010).

**Table 2.** Summary of mouse Y chromosome genes and their known functions. A gene is classified as ‘ancestral’ if it is predicted to have been present in the last common eutherian ancestor. The list of animals where each gene is conserved only includes organisms where a high-quality Y chromosome sequence is available, and the presented list is not exhaustive. *Rbmy was initially thought to be important in spermatid morphogenesis, but this was later questioned (Szot et al., 2003).

Y gene	Sequence class	X-homologue	Y copy number	Conserved in	Reported functions in the testis	Key gene ontology process	Expression pattern
*Sry*	Ancestral	*Sox3*	1	Opossum, bull, rat, mouse, marmoset, rhesus, chimp, human	Testis determination	DNA-binding transcription factor activity	Somatic cells in genital ridge, germ cell specific in adult
*Rbmy array*	Ancestral	*Rbmx*	~30	Opossum, bull, rat, mouse, marmoset, rhesus, chimp, human	Unknown *	RNA splicing	Testis biased
*Zfy1,* *Zfy2*	Ancestral	*Zfx*	2	Bull, rat, mouse, marmoset, rhesus, chimp, human	Apoptotic elimination of univalent spermatocytes at metaphase I, meiosis II completion, MSCI initiation and maintenance, spermatid head and tail morphogenesis	Transcription activator	Spermatogenic cells specific
*Kdm5d*	Ancestral	*Kdm5c*	1	Opossum, rat, mouse, marmoset, rhesus, chimp, human	Unknown	Histone demethylase that specifically demethylates 'Lys-4' of histone H3	Ubiquitous
*Uty*	Ancestral	*Utx*	1	Bull, rat, mouse, marmoset, rhesus, chimp, human	Unknown	Histone demethylase activity (H3-K27 specific)	Ubiquitous
*Ddx3y*	ancestral	*Ddx3x*	1	Bull, rat, mouse, marmoset, rhesus, chimp, human	Unknown	ATP-dependent RNA helicase	Ubiquitous
*Usp9y*	Ancestral	*Usp9x*	1	Bull, rat, mouse, marmoset, rhesus, chimp, human	Unknown	Ubiquitination regulator, peptidase C19	Testis biased
*Uba1y*	Ancestral	*Uba1x*	1	Opossum, bull, rat, mouse	Unknown	U1 ubiquitin activator	Testis biased, mostly spermatogonia and round spermatid
*Eif2s3y*	Ancestral	*Eif2s3x*	1	Bull, rat, mouse	Spermatogonial proliferation and differentiation	Translation initiation	Ubiquitous
*Teyorf*	Acquired	*–*	1	Mouse	Unknown	Claudin transmembrane	Testis specific
*Prslly*	Acquired	*–*	1	Mouse	Unknown	Serine-type endopeptidase activity	Testis specific
*H2al2b, H2al2c*	Acquired	*H2al1*	2	Mouse	Unknown	DNA packaging, pericentric heterochromatin regulation	Testis specific, expressed from round spermatid stage
*Sly*	Acquired	*Slx,* *Slxl1*	126	Mouse	Interacts with SSTY to recruit SMRT/N-Cor in turn mediating spermatid-specific gene expression	Chromatin remodelling	Testis biased
*Srsy*	Acquired	*Srsx*	197	Mouse	Unknown	Unknown	Testis biased
*Ssty1&2*	Acquired	*Sstx*	85&221	Mouse	H3K4me3-reader at the promoter of spermatid-specific genes, recruits SLY and SLX/SLX1	Methylated histone binding	Testis biased
*Rbm31y*	Acquired	*Rbm31x*	2	Mouse	Unknown	RNA binding	Testis biased

**Figure 4.**
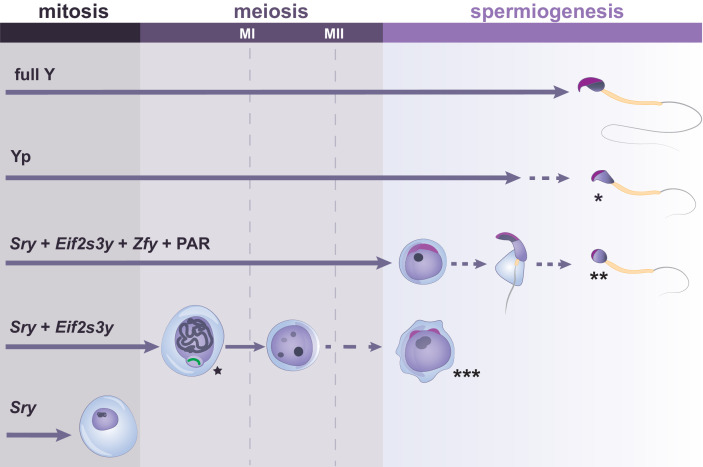
Y-linked gene complements and developmental progression through spermatogenesis. Specific Y genes control the developmental progress of germ cells in spermatogenesis. The presence of the Y short arm (Yp) allows formation of sperm which have abnormal heads, exhibiting reduced curvature and poor chromatin compaction (indicated by *). The presence of *Sry*, *Eif2s3y*, *Zfy* genes and a PAR allows progression to the round spermatid stage, but spermatid elongation is abnormal and delayed, with rare production of sperm with poorly structured heads (indicated by **). Germ cells expressing only *Sry* and *Eif2s3y* have incomplete MSCI (indicated by the star next to the X chromosome shown in green). Moreover, most germ cells of this model arrest before the second meiotic division (MII), with occasional progression to form mostly diploid and abnormally shaped round spermatids (indicated by ***). When only *Sry* is expressed, spermatogonial cells fail to proliferate and to enter meiosis. Arrowed lines show germ cell progression through spermatogenesis, with dotted lines indicating abnormal cell differentiation at a low frequency.

The race to discover the sex-determining region brought spotlight to the Y chromosome. However, the Y chromosome is far from being just a sex switch. It has a clear role in spermatogenesis. Mice with one X chromosome and transgenic expression of *Sry* (XO*Sry*) develop testis populated with pro-spermatogonia that fail to proliferate, leading to absence of meiotic and post-meiotic cells (Figure 4; Mazeyrat et al., 2001). A similar phenotype can be observed in XO germ cells within XO/XY/XYY male mosaic mice (Levy and Burgoyne, 1986).

Furthermore, it seems that additional Y genes are necessary not only in germ cells, but also in testicular somatic cells that are key to support successful spermatogenesis. Indeed, XY spermatogonia transplanted into XX*Sry* testis cannot complete spermatogenesis, whereas segments of immature XY seminiferous tubule transplanted into XX*Sry* testis display normal spermatogenic development of XY germ cells (Ishii et al., 2007). Altogether this highlights the importance of other Y genes besides *Sry* for both somatic and germ cell functions in the mouse testis.

In humans, the first sign that the Y chromosome may be involved in gamete production after testis determination came from the study of sub/infertile men with de novo deletions on the Y (Tiepolo and Zuffardi, 1976). The authors proposed the existence of at least one spermatogenesis factor or ‘azoospermia factor’ (AZF) on the Y. With an increasing number of studies finding sub-fertile males with Y deletions, and with the advances of physical and molecular mapping, three recurrently deleted regions of the human Y long arm were encountered: AZFa, AZFb, and AZFc (Vogt et al., 1996).

By the end of the twentieth century, there was undeniable evidence that both human and mouse Y chromosomes are crucial for spermatogenesis. However, the nature and number of Y-linked genes involved was still completely unknown. The importance of identifying such spermatogenesis factors became clear, especially given their clinical relevance for human fertility.

### The search for spermatogenesis factors

Historically, functional studies of the human Y chromosome have been very challenging. They have relied on genetic analyses of individuals exhibiting vastly different genotypes and fertility phenotypes. A main aim of these heterogeneous studies has been to identify deleted/mutated genes within AZFa, b, and c. However, It has been difficult to draw clear associations between individual genes and specific reproduction phenotypes, especially given the historical lack of good Y assemblies (Colaco and Modi, 2018). Much remains unknown about the role of both ubiquitous and testis-specific genes for human reproduction (Colaco and Modi, 2018). These difficulties have highlighted the need for a genetically tractable model organism to study individual Y-gene functions. For decades, the mouse has been the mammal of choice to study Y-chromosome biology and spermatogenesis. As a result, this review focuses on the biology of the mouse Y chromosome. However, mammalian Ys can be highly divergent, and it is still unclear to what extent findings in the murine model will extend to other mammals.

The realisation that the mouse Y contained key factors for spermatogenesis resulted in attempts to perform loss of function studies using gene knockout. However, classic gene targeting strategies based on homologous recombination in mouse embryonic stem cells, which had worked to study autosomal and X gene functions, proved to be unsuccessful for the Y chromosome. This has been attributed to several factors. Firstly, sequencing data for the Y was sparse, with genomic characterisation relying on incomplete transcription-mapping approaches (Lahn and Page, 1997; Mazeyrat et al., 1998; Soh et al., 2014). The highly repetitive nature of the Y, besides making sequencing difficult using classical approaches, also hindered gene targeting attempts. This limited the field to the use of a few mouse models, which have different deletion variants spanning multiple Y genes, referred as Y-deletants hereafter. By adding transgenes of individual Y genes to the different Y-deletants, researchers investigated the rescue of previously identified spermatogenic failures. Transgenes have also been used in knockdown experiments to express interfering RNAs against ampliconic long arm Y gene (see: Y genes for meiotic progression and spermiogenesis). Overall, transgene rescue strategies, despite some caveats (see: the other indisposable Y genes), have been key to shape our current knowledge of Y-chromosome biology.

### Are only two Y genes essential for male fertility?

In 2001, Mazeyrat et al., 2001 used BAC or cosmid clones of Y genes to generate individual transgenic mouse lines for three single-copy Y genes. These were then bred into two mouse models that express *Sry* but lack most or all of the remaining Y. Whereas Y-deletants experience arrest in spermatogonial proliferation, a clear amelioration was seen for mice expressing an *Eif2s3y* transgene. This ancestral gene has been lost in humans but is ubiquitously expressed in mouse and encodes a subunit of the translation initiation factor Eif2 (Figure 2 and Table 2).

Testes of these ‘rescued’ mice were larger than XO*Sry* controls. Furthermore, the proportion of spermatogonia in rescued mice was similar to that of wild types. Importantly, germ cells progressed to the secondary spermatocyte stage, only arresting before the second meiotic division (Figure 4). Occasionally, spermatid-like cells were observed in these mouse models, although most of them were abnormally shaped and diploid (Figure 4; Mazeyrat et al., 2001; Vernet et al., 2011). Altogether, the authors reported having found the sought-after spermatogonial proliferation factor.

This study would become very influential for later investigations. For instance, Yamauchi and colleagues sought to generate offspring from the sterile mouse models described 13 years prior (Yamauchi et al., 2014). Using assisted reproduction, they were able to fertilise donor oocytes by injecting the rare round spermatids found in the testis of *Sry* and *Eif2s3y-*complemented mouse models. These fertilised oocytes were further transferred into recipient females, leading to the birth of a few offspring. The authors speculated that it may be possible to dispose of the mouse Y chromosome if replacements were made for *Sry* and *Eif2s3y*.

However, the many spermatogenic abnormalities seen when most of the Y chromosome is missing highlight that other Y genes are required for fertility. For instance, Yamauchi and colleagues showed that in mice expressing an additional fragment of the Y chromosome short arm, the abundance of later stage spermatids and the success of assisted reproduction was increased. This stresses the importance of additional Y short-arm genes to complete meiosis and differentiation from spermatid to spermatozoa (Yamauchi et al., 2014). In particular, a family of duplicated paralogues, the zinc-finger Y-linked *Zfy1* and *Zfy2* (Figure 2 and Table 2), has received much attention in the last decade and has been described as a key regulator of multiple spermatogenesis events.

### Y genes for meiotic progression and spermiogenesis

The *Zfy* transcription factors are conserved across eutherians (Table 2). In humans, the single *ZFY* has two splice variants. Contrary to the short variant, the full-length variant has the ability to control transcription via transactivation (Decarpentrie et al., 2012). This seems to be reflected in the mouse, as *Zfy2* expresses the long isoform with higher transactivation activity compared to *Zfy1* (Decarpentrie et al., 2012).

*Zfy* genes are thought to play multiple roles in spermatogenesis. It has been suggested that *Zfy* genes are needed during pachynema for initiation and maintenance of MSCI, as well as apoptotic elimination of cells in which this process is defective (Figures 3 and 4; Vernet et al., 2016a). Later, during the first meiotic metaphase, *Zfy2* regulates the SAC and is required to eliminate cells with unpaired sex chromosomes (Vernet et al., 2011). In Y-deletant models lacking a PAR, sex chromosomes cannot pair, causing spermatocyte univalence. In these models, the checkpoint-mediated apoptosis of univalent spermatocytes fails to take place and aneuploid round spermatids are produced. The addition of a *Zfy2* transgene rescues this phenotype and ensures elimination of univalent spermatocytes (Figure 4; Vernet et al., 2014; Vernet et al., 2011). *Zfy2,* and to a lesser extent *Zfy1,* is also required to promote completion of meiosis II, rescuing the meiotic block seen in Y-deletant models (Figure 4; Vernet et al., 2014). *Zfy2* is also involved in spermatid morphogenesis, as transgenic integration of *Zfy2* in Y-deletants improves spermatid morphology and increases the success rate of assisted reproduction (Figure 4; Yamauchi et al., 2015). Indeed, addition of *Zfy2* contributes to the restructuring of the sperm head and development of the sperm tail (Figure 4; Vernet et al., 2016b). Specifically, these morphogenic changes are thought to be regulated by *Zfy2* through strong expression of the ZFY2 transactivation domain from an alternative, spermatid-specific *Cypt*-derived promoter. However, the molecular mechanisms underlying *Zfy* genes’ functions remain unclear.

In the mouse, the ancestral genes that are mostly conserved across mammals are within the Y chromosome short arm (Figure 2). This partly explains why studies looking for ‘essential’ spermatogenesis factors have mostly focused on this region. However, the Y short arm only represents ~ 2.5% of the full mouse Y length (Figure 2). The rest of the mouse Y euchromatin is encompassed within the long arm, which until 2014 remained mostly un-sequenced (Soh et al., 2014). The mouse Y long arm contains highly repetitive sequences and testis and murine-specific gene clusters consisting of three main gene families: *Sycp3-like Y-linked (Sly*), *Spermiogenesis-specific transcript on the Y (Ssty*) and *Serine-rich Secreted Y-linked (Srsy*) (Figure 2 and Table 2). Since the many copies of these gene families are intermingled (Figure 2), it is very challenging to functionally study *Sly*, *Ssty,* and *Srsy* independently. Indeed, any deletion of one or more amplicon on the Y long arm will encompass all the three main gene families. Y deletant models, which have depletions of all three gene families, show infertility and severe sperm abnormalities (Figure 4), associated with upregulation of spermatid-expressed sex chromosome genes (Conway et al., 1994; Ellis et al., 2005; Styrna et al., 1991; Touré et al., 2004; Yamauchi et al., 2010).

Knockdown of the *Sly* family using transgenic expression of interfering RNAs has shown that SLY depletion is at least partly responsible for the phenotypes seen in long-arm Y deletant mice. Indeed, knockdown of *Sly* causes subfertility and upregulation of spermatid genes (Cocquet et al., 2009; Riel et al., 2013). When knockdown males were able to breed, *SLY* depletion was also linked to female-biased litters. Conversely, knockdown of *Slx/Slxl1,* the X homologues to *Sly,* causes spermatid-specific sex-chromosome gene downregulation, as well as male-biased litters (Cocquet et al., 2012; Cocquet et al., 2010; Kruger et al., 2019). These sex-ratio skews likely result from differential fertilising abilities between X- and Y-bearing sperms in *Sly* and *Slx/Slxl1* depleted models (Kruger et al., 2019; Rathje et al., 2019). Moreover, knockdown of both X and Y families re-establishes correct sex ratio and transcriptional output.

Mechanistically, SLY and SLX/SLXL1 compete for chromatin occupancy at the loci of spermatid-expressed genes, especially on the sex chromosomes (Moretti et al., 2020). This is where SLY is thought to interact with the histone-reader *SSTY* to recruit repressive transcriptional complexes, therefore modulating global spermatid-gene expression (Comptour et al., 2014; Moretti et al., 2020).

Altogether, the pressure to keep a balance in postmeiotic sex-chromosome expression and in sex ratio has created intragenomic conflict between sex chromosomes, with *Sly* and *Slx/Slxl1* acting as antagonistic meiotic drivers (see: Evolutionary forces and functional specialisation of the Y chromosome) (Cocquet et al., 2012; Ellis et al., 2011; Larson et al., 2018). This ‘arms race’ has promoted the amplification of long-arm Y genes and their X homologues shaping the functional and evolutionary dynamic of these loci.

Altogether, the advent of the 21th century has seen an increasing interest in studying Y-linked genes. These efforts have provided valuable information on how some Y genes may participate in the developmental control of spermatogenesis (Figure 4). However, many questions remain. Firstly, even for well-studied genes such as *Eif2s3y* and *Zfy2*, the mechanism of action and molecular interactions of these spermatogenic factors are mostly unknown. Secondly, the majority of Y-linked genes have not yet been assigned to specific spermatogenic function (Table 2). Are they therefore disposable for male fertility?

### The other indisposable Y genes

There is undeniable evidence that additional Y-linked genes are necessary for non-assisted reproduction. Although mice carrying only *Sry, Eif2s3y,* and *Zfy* can produce offspring through assisted reproduction, they are infertile. This is in part due to the lack of the Y chromosome long arm. However, there are other genes on the Y short arm that contribute to spermatogenesis. For instance, mice retaining most of the short arm, but no long arm, develop sperm with better morphology and have increased assisted-reproduction success compared to individuals expressing only *Sry, Eif2s3y,* and *Zfy* complements (Figure 4; Yamauchi et al., 2015).

How should research proceed to characterise the function of these indisposable genes on both the short and long arm of the Y chromosome? Can previously described multi-gene deletion mouse models and transgene strategies still be informative, or has their utility reached a plateau? These time-consuming approaches have been successful but also have inherent limitations to study genes individually. Firstly, transgenes usually integrate at random genomic locations (Yan et al., 2013), which can disturb expression of neighbouring genes. Furthermore, transgenes often integrate in multiple copies and therefore have varying expression levels (Mazeyrat et al., 2001; Royo et al., 2010). These caveats can complicate interpretation of transgene rescue experiments. Even if transgenes are integrated at defined genomic locations, their expression levels can differ from wild-type (Vernet et al., 2016b; Vernet et al., 2014). This is limiting because dosage of ancestral Y genes is thought to be crucial and sensitive for sex-chromosome function (see: Evolutionary forces and functional specialisation of the Y chromosome).

With the inability to perform gene knockouts on the Y, scientists had to make do with transgene-complementation approaches. However, new technologies for gene targeting have been developed, and better-quality Y-chromosome sequence information is now available. Indeed, the rise of gene-editing strategies such as transcription activator-like effector nuclease (TALEN) and CRISPR/Cas9 has opened new avenues for unbiased dissection of Y-gene functions. Targeting of the two most studied Y genes, *Sry* and *Eif2s3y*, recapitulated the expected sex-reversed and spermatogonial-arrest phenotypes, respectively (Kato et al., 2013; Matsubara et al., 2015; Song et al., 2017; Wang et al., 2013; Zuo et al., 2017). These studies provided proof of principles that genome editing was possible on the Y chromosome.

Additionally, other Y chromosome genes have been targeted in the mouse. In some instances, potential spermatogenic functions of targeted Y genes were not investigated in detail. This has been the case for the *Ubiquitously Transcribed Y Chromosome Tetratricopeptide Repeat* (*Uty*) gene. It was shown that this deeply conserved ancestral gene has partially-redundant functions with its X homologue *Utx* during embryonic development (Shpargel et al., 2012; Wang et al., 2013). However, possible spermatogenic abnormalities and subfertility were not examined.

When gene-editing studies of short-arm mouse Y genes have looked at spermatogenic functions, they have faced a major difficulty. Indeed, it has been difficult to ensure complete Y protein abrogation. To date, Y genes have either been targeted at the vicinity of the start codon, e.g. for *Zfy1* and *Zfy2* (Nakasuji et al., 2017); or at an exon not thought to encode a key domain, e.g. *DEAD-Box Helicase 3 Y-Linked (Ddx3y*), *Ubiquitin Specific Peptidase 9 Y-Linked (Usp9y*), *Ubiquitin-like modifier-activating enzyme 1 Y* (*Uba1y*) and *Lysine Demethylase 5D (Kdm5d*) (Matsumura et al., 2019; Zuo et al., 2017). However, we now know that following CRISPR/Cas9 editing, transcription through alternative start codons or skipping of mutated/deleted exons can allow functional protein formation (Lindeboom et al., 2016; Lindeboom et al., 2019 Sharpe and Cooper, 2017; Smits et al., 2019; Wiles, 2020). Because effective antibodies are not currently available for most Y-encoded proteins, it is unclear whether in these deletion models the Y-proteins in question had been completely abolished. As a result, although using nuclease-mediated gene editing is a step in the right direction, only carefully designed strategies ensuring complete protein ablation will help dissect the functions of Y-linked genes.

Although most Y-genes do not yet have ascribed functions, many could be important for spermatogenesis. Non-conserved Y genes that are acquired within mammalian lineages tend to show testis-biased expression, and are therefore predicted to be linked to male fertility (Bellott et al., 2014; Godfrey et al., 2020; Mitchell, 2000; Soh et al., 2014). In the case of the mouse, *Testis expressed, Chromosome Y open reading frame 1 (Teyorf1*), *Protease, serine-like, Y (Prssly*) and *H2A histone family member L2 (H2al2*) (Soh et al., 2014; Figure 2 and Table 2) were discovered only recently, and their functions in fertility have therefore not been investigated.

Ancestral genes are also interesting, because they have been retained on the Y chromosome during evolution and are implicated in critical housekeeping functions. Examples include the ubiquitination proteins *Uba1y* and *Usp9y*, splicing factor *Rbmy*, RNA helicase *Ddx3y* and chromatin modifiers *Uty* and *Kdm5d* (Table 2). Interestingly, some of these ancestral genes seem to have functionally diverged from their X-homologues (Bellott et al., 2014; Snell and Turner, 2018) and evolved testis-biased expression, suggesting a specialised role in spermatogenesis. Preferential testis-expression is observed for *Ddx3y* in primates, *Uba1y* in mice, rats, bulls, and opossums, *Usp9y* in mice and rats and *Rbmy* in bull, rodents and primates (Table 2; Bellott et al., 2014). Furthermore, deletions encompassing *DDX3Y* and *USP9Y* have been recorded in sub-fertile men (Sun et al., 1999; Vogt et al., 2008).

In addition to protein-coding loci, the potential spermatogenic roles of non-coding regulatory regions on the Y chromosome must not be underestimated. Divergence between Y and X chromosome regulatory regions can lead to expression, and potentially phenotypic differences between XY and XX individuals (Godfrey et al., 2020; Naqvi et al., 2019; Naqvi et al., 2018). One could imagine that, just like Y protein-coding genes, Y regulatory regions have experienced pressure to specialise in regulating male fertility, especially through dosage control of nearby Y protein-coding genes.

### Outlook

The Y chromosome has historically been misunderstood. Evolutionary insights and discoveries of spermatogenic factors have shown that the Y chromosome is far from being an ever-degenerating functional wasteland. For a long time, however, technical limitations have restricted the investigation of individual Y gene functions. In an era dominated by fast-evolving gene-editing techniques and omics technologies, the stage is now set for systematic Y-gene deletions and functional characterisation of resulting mutants. Altogether, fully dissecting the link between Y genes and key reproductive processes in the mouse will be paramount to deepen our understanding of genetic components influencing human male fertility and inform us on the functional evolution of the Y chromosome in mammals.

## Acknowledgements

The authors would like to thank members of the Turner lab for providing feedback on the manuscript. This review is dedicated to the memory of Paul Burgoyne.
